# Evaluating the Effect of Sampling Scale on Mosquito Virome Characterization Using PacBio HiFi Long-Read Metagenomics

**DOI:** 10.3390/insects17070721

**Published:** 2026-07-13

**Authors:** Pamela Mancini, David Brandtner, Giulia Cordeschi, Marcello Iaconelli, Valentina Mastrantonio, Daniele Porretta, Giuseppina La Rosa

**Affiliations:** 1 National Center for Water Safety (CeNSiA), Istituto Superiore di Sanità, Viale Regina Elena 299, 00161 Roma, Italy; david.brandtner@iss.it (D.B.); marcello.iaconelli@iss.it (M.I.); giuseppina.larosa@iss.it (G.L.R.); 2 Department of Ecology and Biology, Tuscia University, Via Santa Maria in Gradi 4, 01100 Viterbo, Italy; giulia.cordeschi@unitus.it; 3 Department of Environmental Biology, Sapienza University of Rome, Piazzale Aldo Moro 5, 00185 Roma, Italy; valentina.mastrantonio@uniroma1.it (V.M.); daniele.porretta@uniroma1.it (D.P.)

**Keywords:** mosquito-specific viruses, viral diversity, sample pooling, long-read sequencing

## Abstract

Characterizing the mosquito virome is essential for understanding host–microbiota interactions and vector competence, but it can be influenced by sample scale, sequencing strategy, and host depletion. This study evaluated the effect of sampling scale on mosquito virome characterization using a third-generation sequencing (TGS) metagenomics approach based on PacBio HiFi long reads, applied to L4 larvae and adults of *Aedes mariae*, analyzing single individuals and pools of increasing size before and after host genome removal. The results showed that sequencing yield did not increase with pool size, indicating that the total number of reads is not proportional to the number of individuals. Host genome removal reduced the overall number of reads but altered their composition, increasing the relative proportion of assigned viral reads and reducing unclassified sequences. Despite a similar total read output, virome diversity increased with pool size, with larger pools showing greater taxonomic richness driven by the contribution of each individual. However, the high proportion of unassigned reads suggests the presence of uncharacterized viruses. This methodological workflow was technically feasible for both single-individual and pooled samples. Single-individual analyses may provide complementary information on individual-level virome composition and on low-abundance viral taxa that could be less apparent in pooled samples, whereas pooled samples may facilitate the detection of a broader range of viral taxa and may better capture the shared component of viral diversity within the analyzed population.

## Simple Summary

Viruses associated with mosquitoes can influence both insect biology and disease transmission, yet they remain less studied than the bacterial components of the microbiota. This is partly due to methodological challenges, as different sequencing approaches present complementary characteristics in terms of read length, accuracy, and sequencing output, which may influence virome reconstruction and taxonomic resolution. Developing accurate methods is therefore essential to identify viruses at the species level, especially when only a few individuals are available or when viruses occur at low abundance. This study demonstrates that the virome of a single mosquito can be analyzed using PacBio HiFi sequencing, which produces long and highly accurate reads. This approach may provide valuable information on individual-level virome variability and may facilitate the identification of low-abundance or potentially novel viral taxa that could be underrepresented in pooled samples. Our results suggest that single-individual analysis is technically feasible and may provide complementary information, whereas pooling may facilitate the detection of a broader range of viral taxa. The choice between these strategies therefore depends on the study objectives. Moreover, the high proportion of unassigned sequences indicates that many viruses remain undiscovered, highlighting the importance of accurate methods to explore this hidden diversity.

## 1. Introduction

In recent years, the study of mosquito microbiota has assumed an increasingly central role in understanding the ecology and biology of these insects. Bacteria and viruses constitute key components of their physiology and ecological adaptability [1,2]. However, while the bacterial component has been extensively explored through well-established methodologies, the mosquito virome, defined as the assemblage of viruses associated with mosquitoes [2], remains comparatively understudied, despite its potential role in modulating host fitness and vector competence [3,4,5].

Although most studies on the mosquito virome have historically focused on arboviruses of medical and veterinary importance, the advent of Next Generation Sequencing (NGS) technologies has enabled the identification of numerous Mosquito-Specific Viruses (MSVs), a heterogeneous group of arthropod-restricted viruses that are unable to replicate in vertebrate cells. Despite this host restriction, MSVs are phylogenetically related to several mosquito-borne pathogenic viruses belonging to the families *Birnaviridae*, *Flaviviridae*, *Mesoniviridae*, *Rhabdoviridae*, and *Togaviridae*, as well as to members of the negevirus group and viruses currently classified within the order *Reovirales* [6,7,8,9], suggesting a shared evolutionary history between arthropod-specific viruses and arboviruses.

Evolutionary studies have revealed co-evolutionary relationships between MSVs and their hosts, including the presence of endogenous viral elements integrated into mosquito genomes, while phylogenetic reconstructions have suggested that certain pathogenic bunyavirus lineages may have originated from arthropod-specific ancestors [10]. Collectively, these findings indicate a potential role of MSVs in the evolution and emergence of vector-borne viruses. Moreover, several studies have suggested that mosquito-specific viruses may modulate the replication of medically important arboviruses within mosquito hosts, thereby potentially influencing vector competence and pathogen transmission dynamics [11,12]. Considering that vector-borne diseases account for more than 17% of all infectious diseases worldwide and cause over 700,000 deaths annually [13], understanding the viral diversity associated with mosquitoes is essential not only for public health but also for elucidating the co-evolutionary mechanisms that shape host–microbiota interactions. Achieving this goal, however, requires methodological approaches capable of accurately capturing the complexity of mosquito-associated viral communities. Accurate characterization of mosquito-associated viral diversity, including both dominant and low-frequency viral taxa, therefore requires methodological approaches capable of reliably detecting viruses across a wide range of abundances. In this context, the choice of sampling scale (single individuals versus pools) and sequencing strategy (short-read versus long-read approaches) represents a key factor influencing virome reconstruction and taxonomic resolution [14]. Although the objectives of the study constitute the primary criterion guiding these choices, technical considerations may also play an important role. Both sample structure and sequencing technology may affect virome characterization and should therefore be carefully considered during experimental design.

With regard to sequencing technologies, studies on the mosquito virome have predominantly relied on short-read sequencing approaches using Illumina or MGI platforms [15]. Short-read sequencing studies show marked methodological heterogeneity, with reported read lengths ranging from 150 to 280 bp and total sequencing output varying from approximately 1.5 million to over 10 billion reads per study.

Viral reads generally represent only a small fraction of the dataset (on average 2–9%), and taxonomic assignments are often restricted to relatively broad taxonomic levels, partly due to the complexity of viral communities and the incompleteness of available reference databases [15]. Nevertheless, short-read sequencing remains the most widely adopted approach in metagenomic studies because of its high throughput, accuracy, and cost-effectiveness. Unlike bacterial community profiling, viral metagenomics lacks a universal taxonomic marker. Consequently, taxonomic assignment relies largely on the sequence information contained within each read, making both read length and sequence accuracy crucial factors for virome characterization.

To the best of our knowledge, published mosquito virome studies based on long-read technologies, instead, have so far relied almost exclusively on nanopore-based technologies, particularly Oxford Nanopore Technologies. Although the platform has historically been characterized by higher single-read error rates than short-read sequencing approaches, accuracy has substantially improved with the introduction of the R10.4.1 nanopore and Kit 14 chemistry (>Q20) [16,17]. These studies typically generate reads of approximately 300–500 bp and have enabled the detection of both known and novel viruses. However, they also frequently report low proportions of viral reads (<5–7%) and high fractions of unclassified sequences, particularly when pooled samples are analyzed. Sequencing output varies widely, ranging from fewer than 100,000 to approximately 3 million reads per sample [18,19,20,21]. Consequently, taxonomic assignments remain largely restricted to the family level, although putative species-level assignments have been achieved through increased sequencing depth and assembly-based approaches [18,19,20,21,22]. Overall, these observations highlight the need for long-read metagenomic strategies that combine extended read length with high single-base accuracy, while explicitly evaluating the impact of sampling scale on virome composition and taxonomic resolution.

The present study used the sea-rock pool mosquito *Aedes mariae* as a model system to evaluate a methodological framework based on a third-generation sequencing (TGS) approach using PacBio HiFi long-read technology. PacBio HiFi reads are generated through circular consensus sequencing (CCS), in which multiple passes of the same DNA molecule are combined to produce highly accurate long reads. This technology combines extended read length (>1000 bp) with high base-level accuracy (>Q30) [23], resulting in more complete and detailed target genome sequence information for single reads that may facilitate taxonomic assignment at finer taxonomic scales while complementing established short-read metagenomic approaches [24]. Larval and adult samples of *Aedes mariae*, analyzed as both single individuals and pools of increasing size, were compared to evaluate how sample structure may influence virome characterization and library performance. In this context, the present study was not designed to assess variability through biological replicates; instead, it systematically investigates the effect of sample structure (single individuals vs. pools of increasing size) and host genome removal on virome reconstruction. By focusing on consistent findings observed across experimental conditions, this study aims to provide a methodological framework for optimizing experimental design in mosquito virome research. The results provide practical guidance for selecting sampling and sequencing strategies in future studies of mosquito-associated viral communities, with potential implications for both understanding the ecology and evolution of vector-associated viromes and investigating the mechanisms that influence vector competence and the transmission of vector-borne diseases.

## 2. Materials and Methods

### 2.1. Sample Preparation and Nucleic Acid Extraction

*Aedes mariae* specimens were collected as larvae and pupae from the same supralittoral rock pool in San Felice Circeo, Italy (41°13′18.77″ N, 13°4′5.51″ E) in July 2024 during the reproductive season of the species (mean temperature: 26–28 °C; mean RH: 66–70%). Once in the laboratory, the collected individuals were placed in plastic trays filled with water from their original breeding site and maintained under controlled conditions until adulthood [25]. Adult mosquitoes were collected immediately after eclosion.

Standard morphological taxonomic keys were used for species identification [26]. Prior to analysis, specimens were flash-frozen and stored at −80 °C. Eight samples of *Aedes mariae* were analyzed, including four consisting of fourth-instar (L4) larvae and four consisting of adult females. For each developmental stage, one sample was represented by a single individual, whereas the remaining samples consisted of pools of 2, 3, and 10 specimens, respectively. Prior to analysis, specimens were stored at −80 °C. Eight samples of *Aedes mariae* were analyzed, including four consisting of fourth-instar (L4) larvae and four consisting of adult females. For each developmental stage, one sample was represented by a single individual, whereas the remaining samples consisted of pools of 2, 3, and 10 specimens, respectively. Prior to homogenization and nucleic acid extraction, specimens were treated with 1% sodium hypochlorite for 30 s to remove contaminating microorganisms and rinsed with sterile Milli-Q^®^ water (Merck Millipore, Burlington, MA, USA) for 30 s. Subsequently, the specimens were placed into separate tubes supplemented with 220 μL of Phosphate-Buffered Saline (PBS) and manually homogenized using SP Bel-Art^®^ Proculture Plastic Pestles (SP Bel-Art, Wayne, NJ, USA). To remove insect debris, samples were centrifuged at 16,000× *g* for 5 min at 4 °C, and the supernatant (approximately 200 μL) was collected. Following centrifugation, the supernatant was collected, and the concentration of free DNA and RNA was quantified using a Qubit™ 4 Fluorometer (Thermo Fisher Scientific, Waltham, MA, USA) with the Qubit™ 1X dsDNA High Sensitivity (HS) and Qubit™ RNA High Sensitivity (HS) kits (Thermo Fisher Scientific, Waltham, MA, USA). This step was performed to estimate the amount of extracellular nucleic acids present in each sample and to verify that the amounts of DNase and RNase employed were appropriate relative to the nucleic acid load. Host-derived and environmental extracellular free DNA were then removed using the TURBO DNA-free™ Kit (Thermo Fisher Scientific, Waltham, MA, USA), and free RNA was digested with RNase I (10 U/μL; Thermo Fisher Scientific, Waltham, MA, USA), following the manufacturer’s instructions. These treatments targeted only extracellular nucleic acids, while viral genomes protected within intact viral particles remained unaffected. Following enzymatic treatment, free DNA and RNA concentrations were measured again in the same supernatant before viral nucleic acid extraction, using the same Qubit assays to assess the efficiency of the degradation treatments and confirm the effective removal of extracellular nucleic acids. Viral nucleic acids were subsequently extracted using the automated MagPurix^®^ EVO magnetic-bead extraction system (Zinexts Life Science Corp., New Taipei City, Taiwan) with the Viral/Pathogen Nucleic Acids Extraction Kit B (ZP02012; Zinexts Life Science Corp., New Taipei City, Taiwan), according to the manufacturer’s instructions. Extracted nucleic acids were stored at −80 °C until use. A negative extraction control consisting of molecular biology-grade water (DNase-, RNase-, and protease-free) without biological material was included and processed in parallel with mosquito samples throughout all nucleic acid extraction steps using the same reagents and protocols, in order to monitor potential contamination introduced during sample processing.

### 2.2. Metaviromic cDNA Preparation, Sequence-Independent Single Primer Amplification (SISPA) and Sequencing

Metaviromic cDNA was generated using the Sequence-Independent Single Primer Amplification (SISPA) technique, following Moreno & O’connor 2020 [27], with minor modifications, which are described in detail below. Reverse transcription and second-strand cDNA synthesis (SISPA A: Reverse Transcription and 2nd Strand cDNA Synthesis—Primer A Addition) were performed using the SuperScript IV First-Strand Synthesis System (Thermo Fisher Scientific, Waltham, MA, USA) in combination with Primer A (5′-GTTTCCCACTGGAGGATA-N9-3′). Subsequently, second-strand synthesis was carried out using Sequenase DNA polymerase (Affymetrix, Santa Clara, CA, USA). This procedure allowed the simultaneous processing of RNA viruses (converted to cDNA) and DNA viruses present in the sample. Prior to amplification, a size-selection step was performed using MagSi-NGSPREP Plus magnetic beads (Magtivio B.V., Nuth, The Netherlands) at a bead-to-sample ratio of 0.55× with the aim of depleting short fragments approximately below 500–700 bp that could interfere with long-read sequencing. The cDNA and DNA amplification step (SISPA B: PCR Amplification of Randomly Primed cDNA and DNA) was performed using AccuTaq LA DNA polymerase (Sigma-Aldrich, Poole, UK) and 1 μL of Primer B (5′-GTTTCCCACTGGAGGATA-3′, 100 pmol/μL). The modified PCR conditions were as follows: initial denaturation at 98 °C for 30 s, followed by 30 cycles of 94 °C for 15 s, 50 °C for 20 s, and 68 °C for 5 min, with a final extension at 68 °C for 10 min. Amplified products underwent an additional purification and size-selection step using MagSi-NGSPREP Plus beads (Magtivio B.V., Nuth, The Netherlands) at a 0.55× ratio. DNA concentration was then quantified using the Qubit™ 4 Fluorometer with the Qubit™ 1X dsDNA High Sensitivity (HS) and Qubit™ 1X dsDNA Broad Range (BR) (Thermo Fisher Scientific, Waltham, MA, USA). The negative control from the extraction step, subsequently subjected to SISPA, and a no-template control included during SISPA amplification were processed in parallel with the samples using identical reagents and experimental conditions in order to monitor potential contamination introduced during sample processing. Library preparation was performed by the sequencing service Macrogen using the SMRTbell Prep Kit 3.0 (Pacific Biosciences, Menlo Park, CA, USA). Libraries underwent the standard technical quality control procedures implemented by the sequencing service. Sequencing was carried out on the PacBio Revio platform (Pacific Biosciences, Menlo Park, CA, USA) to generate HiFi reads. According to PacBio technical specifications, libraries of approximately 1–1.8 kb can generate approximately 10–13 million HiFi reads per Revio SMRT Cell, depending on library quality and loading efficiency.

### 2.3. Host Depletion

Viral integrations, also known as endogenous viral elements (EVEs), are viral sequences integrated into the mosquito genome that may be erroneously classified as viral reads during metagenomic analyses. Such integrations have been documented in different *Aedes* mosquito species [28,29]. Therefore, the sequencing data were analyzed both before and after host genome removal to assess the extent to which host-derived sequences may influence virome characterization. To reduce host-derived background, a host-depletion step was performed by aligning HiFi long reads against a mosquito host reference genome and removing reads with reliable host alignments. The host reference was downloaded from NCBI (assembly accession GCF_002204515.2, AaegL5.0). As no reference genome is currently available for *Aedes mariae*, the chromosome-level *Aedes aegypti* AaegL5.0 assembly was selected as the most complete and well-annotated chromosome-scale reference genome currently available within the genus *Aedes* for host-read depletion. The genomic FASTA file (GCF_002204515.2_AaegL5.0_genomic.fna) was used to build a minimap2 index (.mmi) [30]. Reads were aligned to the host reference using minimap2 v2.26-r1175 [30] with the PacBio HiFi preset (-ax map-hifi). Alignments were processed with SAMtools v1.20 [31] to retain only mapped primary alignments, excluding unmapped reads (flag 0x4), secondary alignments (flag 0x100), and supplementary/chimeric alignments (flag 0x800) (i.e., SAMtools view-F 2308). In addition, a CIGAR-based soft-clipping filter was applied to remove partial/unstable mappings: for each primary alignment, the fraction of soft-clipped bases (S) relative to the query length (computed from the CIGAR operations consuming the query: M/I/S/ = /X) was calculated, and alignments with soft clipping >5% were discarded. Read identifiers passing the host-mapping criteria were exported and used to remove the corresponding sequences from the original FASTQ, generating a final non-host FASTQ file for downstream metagenomic analysis. Intermediate files were retained for reproducibility (sorted BAM of host alignments, primary-only BAM, soft-clip–filtered BAM, and host read ID list).

### 2.4. Bioinformatic Analyses and Pipeline Development

Fastq files were analyzed both before and after host genome removal, and were used in their entirety as input for taxonomic assignment of individual reads through protein-space matching using the Kaiju v1.10.1 tool [32] with default parameters and using only the viruses database (Only viruses from the NCBI RefSeq database—year 2024). Taxon assignment was considered valid by applying a threshold of at least 5 reads. After taxonomic assignment, the resulting read classifications were used to derive descriptive ecological information on virome composition, including virome richness, relative abundance, and overall virome diversity, as well as to distinguish the core and accessory components of the virome. For clarity, the operational meaning of these terms as applied in the present study is provided below. Virome richness is defined as the number of distinct viral taxa detected per sample, whereas relative abundance is calculated as the proportion of reads assigned to each viral taxon over the total number of classified viral reads, expressed as a percentage [33,34,35]. Instead, the term virome diversity is used in a descriptive sense to indicate the overall complexity of the viral community detected in each sample, reflecting the variety of viral taxa present and their distribution in terms of presence, without implying the computation of formal diversity indices [36]. The terms core virome and accessory virome are also used in a descriptive sense. The core virome refers to viral taxa that are consistently detected across samples and remain stably represented regardless of developmental stage or pooling strategy, typically accounting for the most abundant and recurrent components of the community. In contrast, the accessory virome denotes viral taxa detected intermittently or at low prevalence, often individual-specific and occasionally unassigned, which contribute to the broader variability and “rare” component of the virome. These terms are thus used to describe compositional patterns rather than to indicate formally quantified core/accessory partitions [37,38].

## 3. Results

### 3.1. Quantification and Quality Analysis of Nucleic Acids at the Different Stages of Preparation

The nucleic acid concentration data illustrate, for all samples analyzed, three key stages of the workflow: the amount of free extracellular DNA and RNA present before enzymatic depletion of host-derived and environmental nucleic acids, the residual free nucleic acids remaining after enzymatic treatment, and finally the concentration and characteristics of the amplified viral fraction following viral nucleic acid extraction, cDNA synthesis, and sequence-independent single-primer amplification (SISPA) (Table 1).

Total DNA and RNA concentrations progressively increase with pool size in both larval and adult stages, consistent with the greater amount of biological material. After removal of host nucleic acids, however, viral DNA and RNA concentrations drop below the detection limits of the Qubit™ assays (“too low”) in almost all samples; the only exception is the pool of ten larvae, which retains a quantifiable viral DNA concentration. This result indicates that the residual viral fraction is generally insufficient for direct use in subsequent analytical steps. Following cDNA synthesis and SISPA, all samples display measurable concentrations of viral DNA/cDNA, with values that tend to increase with pool size, although not in a strictly linear manner. Amplicon sizes are overall consistent across samples, ranging between 1055 and 1222 bp, with no evident differences attributable to developmental stage or pool size. Electropherograms representing the fragment-size distributions of cDNA/DNA, generated by capillary gel electrophoresis with the Agilent Femto Pulse system, are presented in Supplementary Material Figure S1 for L4 larvae and adult samples. The extraction blank processed in parallel with the samples and subsequently subjected to SISPA amplification yielded only extremely low DNA concentrations and short fragments (~100 bp). Consequently, library preparation was unsuccessful and no sequencing reads were generated.

**Figure 1:**
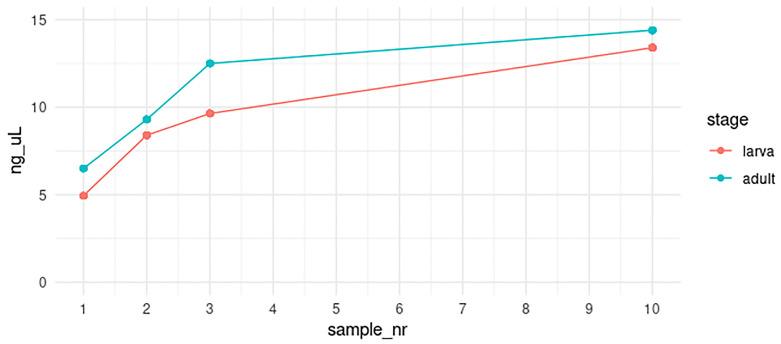
cDNA/DNA (ng/µL) yield curve as a function of pool size in larval and adult samples.

Figure 1 illustrates the increase in cDNA/DNA concentration (ng/µL) as a function of the number of individuals included in each pool, for both larval and adult mosquitoes.

The most pronounced difference is observed between single individuals and pools of three: in larvae, cDNA/DNA concentration increases from 4.94 ng/µL (1 individual) to 9.64 ng/µL (3 individuals), corresponding to a 95.1% increase, while from 3 to 10 individuals, the increase is more moderate (+39.0%). In adult samples, cDNA/DNA concentration increased from 6.5 ng/µL (1 individual) to 9.3 ng/µL (3 individuals) (+43.1%). Although the pool size increased from 3 to 10 individuals, cDNA/DNA concentration increased by only 54.8%, indicating a diminishing return in nucleic acid yield with increasing pool size. In both larvae and adults, cDNA/DNA yield does not increase linearly with pool size, and the curves progressively approach a plateau.

### 3.2. Metagenome Amplicon Sequencing

All sequencing reads are archived in the NCBI Sequence Read Archive and organized under the unified BioProject ID PRJNA1406307. Metrics describing both sequencing yield and the characteristics of the PacBio HiFi reads are reported in Table 2:

The HiFi read bases, representing the total number of high-accuracy nucleotide bases produced for each sample, show substantial variability across the dataset, ranging from approximately 383 million (sample 8) to 839 million (sample 2). The number of HiFi reads also varied across samples, ranging from 533,481 reads (sample 8) to 852,147 reads (sample 2). The HiFi N50, reflecting the typical length of the longest reads contributing most of the data, ranged from 756 bp (sample 8) to 1071 bp (sample 2). Mean read lengths were consistent with the N50 values, ranging between 718 bp and 985 bp. Average read quality was high and consistent across all samples (Q34–Q35), in line with expected PacBio HiFi performance. Finally, the average number of passes per molecule ranged from 32 to 39, indicating robust consensus generation across libraries [23,39].

### 3.3. Bioinformatic Analysis

The results of the bioinformatic analysis for the eight samples, both before and after bioinformatic host-read removal, are summarized in Table 3, including the total, assigned, and unassigned reads. Detailed information for each sample, such as taxonomic assignments, the number of reads attributed to each taxon, and their relative abundances before and after host genome removal, is provided in the Supplementary Material Tables S1–S3.

Sequencing yield did not increase linearly with pool size in either larval or adult samples, both before and after host-read removal. Host filtering reduced the overall number of reads by removing sequences mapping to the host genome, affecting both assigned and unassigned reads. Despite this reduction, the proportion of viral reads generally increased after filtering, whereas the fraction of unclassified reads decreased in both larval and adult samples. Within the analyzed dataset, higher proportions of assigned viral reads were generally observed in some pooled samples compared with single-individual samples. In both larvae and adults, pools of three individuals showed the highest proportion of assigned viral reads after host genome removal (39.37% and 36.62%, respectively), whereas single-individual samples were characterized by higher proportions of unclassified sequences. Notably, the adult single-individual sample produced a high number of reads but showed very low viral assignment (<1%) and a predominance of unclassified sequences (>99%). Overall, some pooled samples contained a broader range of detectable viral taxa and a more complex virome composition than single-individual samples, both before and after host genome removal. This trend was reflected by the higher numbers of viral taxa detected in larger pools, which reached up to 17 viral families and 125 viral species in larval samples and 17 viral families and 66 viral species in adult samples. However, because each experimental condition was represented by a single sample, these observations should be considered descriptive trends within this dataset rather than evidence of general biological patterns. Host filtering resulted in a modest reorganization of taxonomic profiles, generally leading to slight reductions in the number of identified species while maintaining a similar number of viral families. The highest taxonomic richness was observed in pooled samples, reaching up to 17 viral families and 125 viral species in larval samples and 17 viral families and 66 viral species in adult samples. Overall, host filtering preserved the general taxonomic structure of the virome while reducing the number of unclassified reads and increasing the relative proportion of assigned viral sequences.

**Figure 2:**
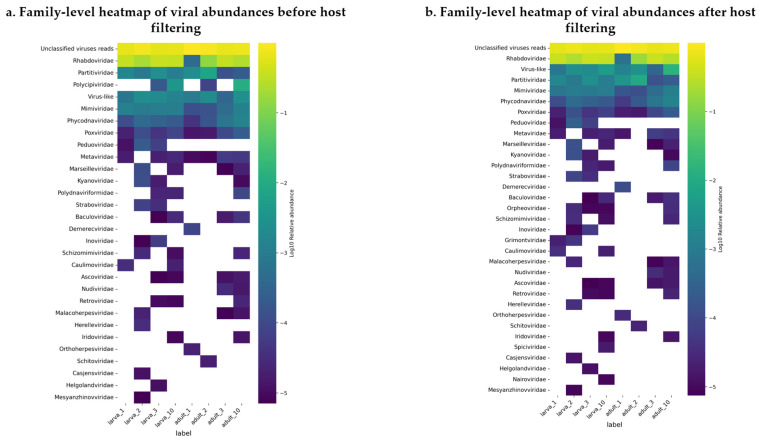
Heatmap of the abundances of all viral families for each sample (scale: log_10_ relative abundance), before and after host filtering.

**Figure 3:**
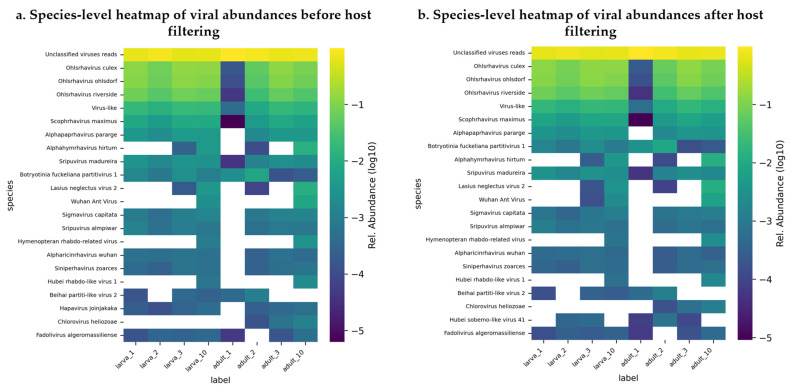
Heatmap of top 20 ranked assignments across samples at species level (scale: log_10_ relative abundance) before and after host filtering.

Relative abundances of all detected viral families and the top 20 species-level assignments across all samples are shown in Figure 2 and Figure 3, respectively, comparing data before and after host genome removal and illustrating differences in viral composition between larval and adult samples and across pool sizes.

A detailed comparison, including percentages of removed reads and assigned/unassigned reads for major viral taxa, is provided in Supplementary Material Table S3.

Figure 2 shows that, both before (2a) and after host genome removal (2b), the virome is dominated by members of the *Rhabdoviridae* family, followed by *Partitiviridae*, *Mimiviridae*, and *Phycodnaviridae*, which are present across all samples with variable abundances. Host genome removal results in a reduction in reads across all viral categories (Supplementary Material Table S3), but does not substantially alter the overall virome structure. In all samples, the fraction of unassigned reads remains predominant, although it decreases after filtering (6–31%). “Reads assigned to viruses” and the “virus-like” category both show limited reductions (1–4% and 1–6%, respectively). It should be specified that the “virus-like” category includes reads classified by Kaiju [32] as showing significant protein-level similarity to viral reference sequences, but lacking sufficient taxonomic resolution for confident assignment to a recognized viral family, genus, or species. Among the assigned viral families, reductions are generally modest and variable across samples: *Partitiviridae* (0.3–0.7%), *Rhabdoviridae* (2–6%), *Phycodnaviridae* (0–10%), and *Mimiviridae* (0–44%). The latter shows the greatest variation, with a maximum reduction of 44% observed in the single larval (L4) sample, whereas no reduction is observed in the single adult sample.

Figure 3 shows that, within the analyzed dataset, the species-level viral composition remained broadly similar across the different pool sizes and between conditions before (3a) and after (3b) host genome removal, with several species detected in both larval and adult samples. Among these, *Ohlsrhavirus culex*, *Ohlsrhavirus ohlsdorf*, *Ohlsrhavirus riverside*, *Scophrhavirus maximus*, and *Sripuvirus madureira* are the most abundant and recurrent across all samples. Host genome removal does not substantially alter this dominant core, with limited but variable reductions: 1–4% for *Ohlsrhavirus culex*, 1–3% for *Ohlsrhavirus ohlsdorf*, 1–18% for *Ohlsrhavirus riverside*, and 0–10% for *Scophrhavirus maximus*.

Within this dataset, pools of three and ten individuals contained a larger number of reads assigned to the most abundant viral species than the corresponding single-individual samples. Alongside this core group, numerous additional species are detected at low abundance and with discontinuous distribution across samples, representing the most variable fraction of the virome. After host genome removal, some of these low-abundance species become less represented or disappear, suggesting a reduction in marginal assignments. The fraction of unassigned reads remains predominant but decreases after filtering (6–31%). Overall, these observations suggest that species-level virome composition was broadly preserved following host genome removal. While variation in viral composition was observed among samples, differences between the corresponding pre- and post-filtering conditions appeared limited. Filtering also leads to a slight reduction in “reads assigned to viruses” (1–4%) and in “virus-like” sequences (0–9%).

## 4. Discussion

The present study evaluated how sample structure (single individuals versus pools of increasing size) may influence mosquito virome characterization using a PacBio HiFi long-read metagenomic workflow. Previous studies have highlighted that the choice between individual and pooled mosquito samples can influence microbiome and virome reconstruction, with each strategy presenting specific advantages and limitations [14,40,41]. Although short-read sequencing remains the most widely adopted approach in viral metagenomics, long-read technologies may provide complementary advantages for virome characterization [42,43]. Because viral metagenomics lacks a universal taxonomic marker, taxonomic assignment relies largely on the sequence information contained within individual reads. Consequently, long-read metagenomic strategies that combine extended read length with high single-base accuracy may facilitate virome reconstruction and taxonomic assignment at finer taxonomic scales.

In the present study, a PacBio HiFi-based metagenomic approach was applied to the larval and adult stages of *Aedes mariae* to evaluate how sampling scale may influence virome characterization and the level of taxonomic assignment achieved, as well as the efficiency of metaviromic library preparation. The study was not designed to assess intra-condition variability through biological replicates, but rather to compare different sample structures (single individuals versus pools of increasing size) and evaluate the potential impact of host genome removal on virome reconstruction. Since our specimens were caught from the wild, some degree of individual biological variability related to physiological status, developmental history, or virome composition cannot be excluded. However, the specimens used in this study originated from the same supralittoral rock pool and were collected during the same reproductive season, which limits the environmental heterogeneity among samples. Furthermore, adult mosquitoes were obtained under controlled laboratory conditions from field-collected specimens. These measures were adopted to minimize potential environmental sources of variation among samples and to reduce the influence of external confounding factors. Therefore, although the results presented here should be interpreted primarily as methodological observations because each experimental condition was represented by a single sample, they offer preliminary methodological insights for optimizing experimental design in mosquito virome research.

### 4.1. Sampling Scale and Nucleic Acid Yield

DNA and RNA concentrations increased with sample size prior to RNase and DNase treatment, reflecting the greater biomass associated with larger pools. However, the drastic reduction in extracellular nucleic acids following host nucleic acid removal indicates that the extracellular viral fraction represents only a minor component of the total nucleic acid content, confirming that in mosquitoes the viral signal is strongly masked by host-derived material [44,45]. This finding highlights the importance of combining depletion strategies with a specific amplification step to render the virome accessible to metagenomic analysis. In this context, SISPA proved to be a crucial step, enabling the recovery of quantifiable viral DNA and cDNA in all samples, including single individuals. It should be noted, however, that SISPA is an amplification-based approach and may introduce biases in the representation of individual viral taxa. Therefore, the relative abundance of reads observed after amplification should not be interpreted as a direct estimate of the original viral abundance present in the samples, but rather as a representation of the amplified virome. Within this dataset, the increase observed after amplification was not proportional to the number of individuals included in each pool. For example, the largest increase in nucleic acid concentration was observed between single-individual samples and pools of three individuals, whereas more limited differences were observed among the larger pool sizes. Within this dataset, increases in amplified viral material appeared progressively less pronounced in the larger pools, suggesting a possible tendency towards a plateau in yield. From a methodological perspective, the fact that a single individual already provides sufficient material for constructing libraries compatible with PacBio HiFi sequencing demonstrates the high sensitivity of the workflow. In addition, pools of three individuals yielded some of the highest post-amplification concentrations observed in this dataset while maintaining a relatively limited sample complexity.

The high homogeneity of amplicon sizes after SISPA, independent of developmental stage or pool size, further highlights the high reproducibility of the protocol. The observed homogeneity of amplicon size distributions, achieved through the combined effect of SISPA efficiency and a double size-selection step, is particularly relevant across different long-read sequencing platforms, as it minimizes technical bias during library preparation and sequencing and ensures greater comparability among samples. Overall, these results indicate that workflow quality and robustness depend not on the amount of starting material, but on the optimization of enrichment and library preparation steps. Quality metrics of HiFi reads further indicate that the workflow is robust and reproducible regardless of input material. Although the total number of reads varies among samples, read quality remains consistently high (Q34–Q35), confirming the suitability of the protocol even for low DNA inputs.

### 4.2. Sampling Scale and Virome Resolution

Sequencing yield did not increase linearly with pool size in either larval or adult samples. For example, in larvae, 681,388 reads were obtained from a single individual compared with 563,682 reads from a pool of ten individuals. Within the analyzed dataset, sequencing output did not increase proportionally with the number of individuals included in each sample and may have been influenced by factors related to sample and library preparation. Importantly, the observed plateau is unlikely to reflect saturation of the PacBio HiFi sequencing platform. The Revio system can generate approximately 10–12 million HiFi reads per SMRT Cell for libraries of comparable size, whereas the total sequencing output obtained in this study remained substantially below this capacity. Within the analyzed dataset, the inclusion of additional individuals was not consistently associated with the detection of additional viral taxa, whereas dominant viral taxa remained well represented across pool sizes. One possible explanation is that low-abundance or individual-specific viruses may contribute less to the overall virome profile in pooled samples, where dominant taxa account for a larger fraction of the assigned reads.

Host filtering results in an overall reduction in read counts, as sequences mapping to the host genome are removed. This highlights the substantial impact of host-derived material, which may persist despite DNase and RNase treatment and is effectively removed only through bioinformatic processing. Because no reference genome is currently available for *Aedes mariae*, host depletion was performed using the chromosome-level *Aedes aegypti* AaegL5.0 assembly, which represents the most complete and best-annotated chromosome-scale reference genome currently available within the genus *Aedes*. Consequently, a fraction of host-derived sequences may have remained unfiltered, potentially contributing to the residual unclassified fraction and to some virus-like assignments. Therefore, post-sequencing host genome removal represents a crucial step for improving the accuracy of virome taxonomic assignment.

Regarding viral composition, despite the reduction in total reads after filtering, their distribution shifts: the proportion of reads assigned to viruses increases, while unclassified reads decrease in both larvae and adults. Although total read counts did not scale proportionally with pool size, samples containing larger numbers of individuals generally contained a greater number of detected viral taxa within this dataset. Within this dataset, larger pools contained higher numbers of viral families and species than single-individual samples. The pools of three and ten individuals showed the highest proportions of assigned reads among the analyzed samples, which further increased after host removal (e.g., from 38.25% to 39.37% in larvae and from 33.85% to 36.62% in adults), whereas lower values were observed in smaller pools and in single individuals, particularly in adults (0.42–0.60%). In the analyzed samples, lower proportions of unclassified reads were observed in some of the larger pools, although this fraction remained high across all samples and comparable between family- and species-level assignments. The similarity of this fraction between family and species levels indicates that increasing taxonomic resolution did not substantially improve assignment efficiency within the analyzed dataset, suggesting that a significant proportion of sequences lack matches in reference databases or belong to previously undescribed viruses.

The reduction in the fraction of reads initially classified as viral after host filtering suggests that some may be host-derived but share similarity with viral sequences. This may be explained by the presence of homologous regions between host and viral genomes, including endogenous viral elements (EVEs) derived from ancient viral integrations [46,47,48]. Additionally, coevolutionary processes may increase similarity between host and viral genomes, contributing to potential ambiguities in taxonomic assignment [49]. Consistent with these evolutionary dynamics, it has also been shown that virome composition varies primarily with mosquito species, reflecting a strong host–virus evolutionary association [50]. These observations are consistent with the presence of commonly detected taxa alongside a more variable fraction composed of low-abundance and sample-specific assignments. Pooling appeared to increase the relative representation of taxa shared among individuals, while some low-abundance taxa were less evident in pooled samples. In contrast, some putative low-abundance or potentially novel viral assignments were observed only in individual samples, particularly among adults. These findings suggest that individual and pooled sampling strategies may provide complementary information depending on the objectives of a study. From a methodological perspective, pooling samples offers several practical advantages, including increased biomass availability, which may improve nucleic acid recovery and reduce the relative influence of contamination [14,41,51]. Pooling also enables the analysis of a larger fraction of a population while reducing the number of samples requiring sequencing, thereby lowering overall sequencing costs [14]. Conversely, individual-level analyses preserve information on inter-individual variation and may facilitate the observation of biological variability that could be masked in pooled samples [14,40,41]. However, additional studies including biological replication will be required to confirm the reproducibility of these observations and the biological relevance of such assignments. Our results also indicate that sequencing of single individuals is technically feasible for viral metagenomics, consistent with findings by Shi et al. (2019) [41], who reported no significant differences between single and pooled samples. That study also identified a stable shared virome alongside marked inter-individual variability and reported putative novel viral taxa, highlighting the value of individual-level analyses. However, those findings were based on short-read technologies. In contrast, the PacBio HiFi approach applied in this study combines extended read lengths (>1000 bp) with high base-level accuracy (>Q30). Thus, unlike traditional long-read technologies, it provides single reads that retain an accuracy comparable to short-read sequencing while containing substantially more target genome sequence information. This feature may facilitate taxonomic assignment at finer taxonomic scales while complementing established short-read metagenomic approaches for virome characterization. This improves taxonomic assignment reliability, reduces the risk of chimeric sequences, and enhances the detection of divergent or previously undescribed viruses, overcoming key limitations of short-read approaches. The resulting reads, with an average length exceeding 1000 bp, provide greater informational content and support taxonomic assignments at a finer taxonomic scale. This is particularly important given that the unassigned read fraction represents the predominant component of the virome and likely harbors novel or highly divergent viral sequences. In this context, the use of highly accurate sequencing technologies is essential to improve the characterization of this largely unexplored component. In the present dataset, a substantial proportion of reads remained unclassified even after host-read removal, highlighting both the potential presence of poorly characterized viral taxa and the current limitations of available viral reference databases. Because each experimental condition was represented by a single sample, the observations discussed above should be interpreted as methodological observations derived from this pilot dataset rather than as evidence of general biological patterns.

## 5. Conclusions

This study highlighted the robustness and effectiveness of the PacBio HiFi–based workflow, which generated long, high-quality sequences across all analyzed samples, including those characterized by low DNA input. The depletion of host- and environmental-derived free nucleic acids prior to nucleic acid extraction, together with the subsequent bioinformatic filtering of host-derived sequences after sequencing, was associated with improved viral sequence identification within the analyzed dataset. The results obtained in this pilot methodological study indicate that single-individual samples can provide sufficient material for virome characterization and may facilitate the detection of viral taxa that are less evident in pooled samples. In contrast, pooled samples showed a broader representation of the viral taxa detected within this dataset, although some low-abundance taxa appeared less represented. These observations suggest that individual and pooled sampling strategies may provide complementary information, depending on the specific objectives of a study. Single-individual analyses may be useful for investigating individual-level variation and identifying rare or previously undescribed viral taxa, whereas pooled samples may be advantageous for obtaining a broader overview of the viral taxa detected within a group of individuals. The high proportion of unassigned reads highlights current limitations of available reference databases and suggests the presence of uncharacterized viruses. Finally, although the method demonstrated considerable potential in mosquitoes, its application could be extended to other organisms and ecological contexts, provided that further validation is conducted in different biological systems.

## Figures and Tables

**Table 1:** Quantification of nucleic acids at different stages of the *Aedes mariae* sample preparation process.

		Before Viral Nucleic Acids Extraction				After Viral Nucleic Acids Extraction	
ID	Nnumber. and Stage of Individuals	Concentration of Free Nucleic Acids		Concentration of Residual Free Nucleic Acids After Degradation Treatment		Concentration of Viral Fraction After cDNA Synthesis and Amplification	
		DNA ng/µL	RNA ng/µL	DNA ng/µL	RNA ng/µL	cDNA/DNA ng/µL	Amplicon Size (bp)
1	1 L4 larva	0.0075	0.0152	too low	too low	4.94	1164
2	2 L4 larvae	0.0170	0.0268	too low	too low	8.4	1221
3	3 L4 larvae	0.0288	0.0373	too low	too low	9.64	1106
4	10 L4 larvae	0.0654	0.0402	0.0244	too low	13.4	1055
5	1 adult	0.0059	0.0205	too low	too low	6.5	1222
6	2 adults	0.0068	0.0292	too low	too low	9.3	1154
7	3 adults	0.0087	0.0308	too low	too low	12.5	1123
8	10 adults	0.0388	0.0441	too low	too low	14.4	1138

**Table 2:** Quality and quantity metrics of HiFi reads.

Sample ID	Number and Stage of Individuals	HiFi Read Bases	HiFi Reads	HiFi N50	Average Read Length	Average Read Quality	Average Pass
1	1 L4 larva	598.975.100	681.388	972	879	Q35	33
2	2 L4 larvae	839.402.537	852.147	1.071	985	Q35	32
3	3 L4 larvae	609.253.385	703.135	944	866	Q35	34
4	10 L4 larvae	426.860.221	563.682	801	757	Q34	38
5	1 adult	740.443.891	804.214	1.020	920	Q35	32
6	2 adults	502.320.411	636.771	866	788	Q35	33
7	3 adults	525.557.884	664.175	851	791	Q35	34
8	10 adults	383.046.038	533.481	756	718	Q34	39

**Table 3:** Metadata and taxonomic assignments of reads, including counts at the family and species levels, before and after bioinformatic removal of host-derived reads.

ID Sample	Stage of Individuals	N° of Individuals	N° of Total Reads	Reads of Unclassified Viruses		Reads Assigned to Viruses		Viral Families	Viral Species
**Before bioinformatic host-read removal**									
			n°	n°	%	n°	%		
1	L4 larva	1	681.388	443.964	65.16	237.424	34.84	8	44
2	L4 larva	2	852.147	673.914	79.08	178.233	20.92	15	79
3	L4 larva	3	703.135	434.177	61.75	268.958	38.25	16	84
4	L4 larva	10	563.682	364.657	64.69	199.025	35.31	15	125
5	adult	1	804.214	800.846	99.58	3.368	0.42	8	17
6	adult	2	636.771	543.129	85.29	93.642	14.71	8	49
7	adult	3	664.175	439.322	66.15	224.853	33.85	11	52
8	adult	10	533.481	391.052	73.30	142.429	26.70	17	66
**After bioinformatic host-read removal**									
			n°	n°	%	n°	%		
1	L4 larva	1	624.454	390.732	62.57	233.722	37.43	9	43
2	L4 larva	2	709.587	534.028	75.26	175.559	24.74	17	76
3	L4 larva	3	671.332	407.021	60.63	264.311	39.37	16	83
4	L4 larva	10	535.087	341.451	63.81	193.636	36.19	17	56
5	adult	1	554.163	550.827	99.40	3.336	0.60	8	17
6	adult	2	487.470	397.758	81.60	89.712	18.40	6	43
7	adult	3	599.805	380.178	63.38	219.627	36.62	11	50
8	adult	10	490.247	353.305	72.07	136.942	27.93	17	62

## Data Availability

The sequencing reads generated in this study are deposited in the NCBI Sequence Read Archive under the unified BioProject accession number ID PRJNA1406307.

## Supplementary Materials

The following supporting information can be downloaded at: https://www.mdpi.com/article/10.3390/insects17070721/s1, Figure S1: Electropherograms of cDNA/DNA Fragment-Size Distributions; Table S1: Taxonomic assignments before host depletion; Table S2: Taxonomic assignments after host depletion; Table S3: Summary of read counts for the most abundant viral families and species before and after host depletion.
