# Supplementary material for: Evaluating the Effect of Sampling Scale on Mosquito Virome Characterization Using PacBio HiFi Long-Read Metagenomics
Source: Insects. 2026 Jul 13;17(7):721. doi: 10.3390/insects17070721 (PMC13410176; doi:10.3390/insects17070721)
Supplement: Supplementary file 1 [file insects-17-00721-s001.zip › Figure S1_Electropherograms of cDNA_DNA Fragment-Size Distributions.pdf]

Additional file 1: Supplementary Figure S1

ID 1: 1 individual L4 larva

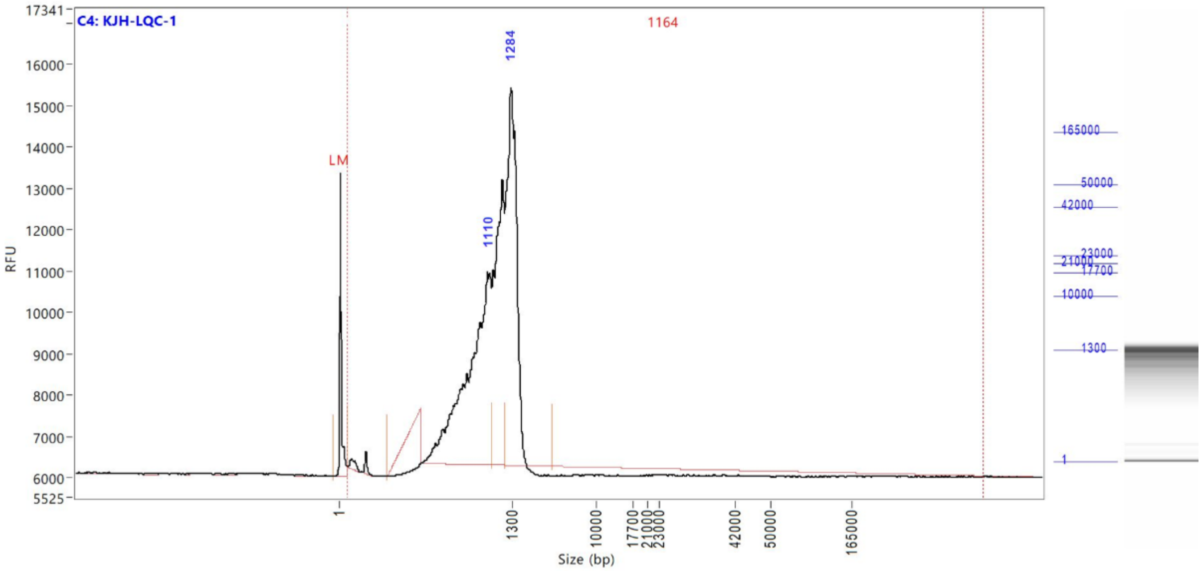

ID 2: 2 L4 larval individuals

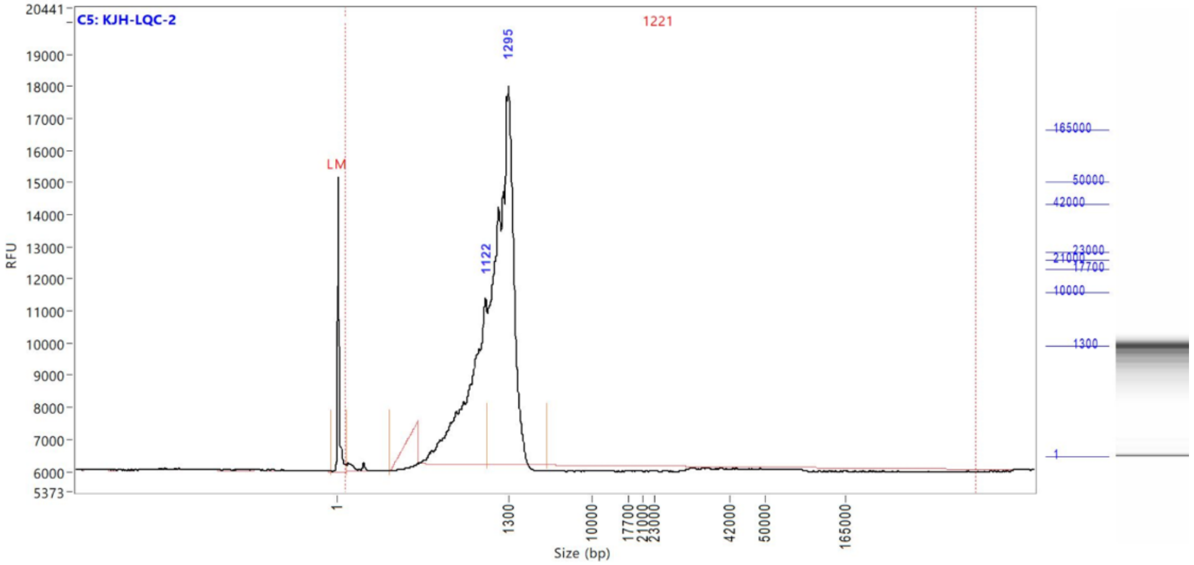

ID 3: 3 L4 larval individuals

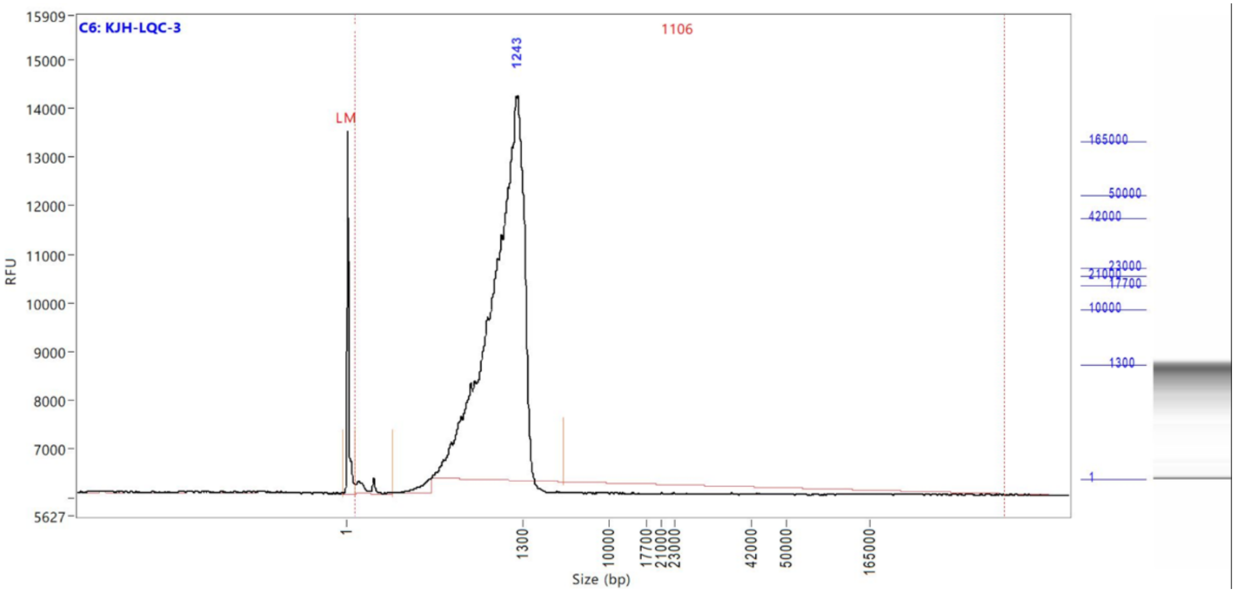

ID 4: 10 L4 larval individuals

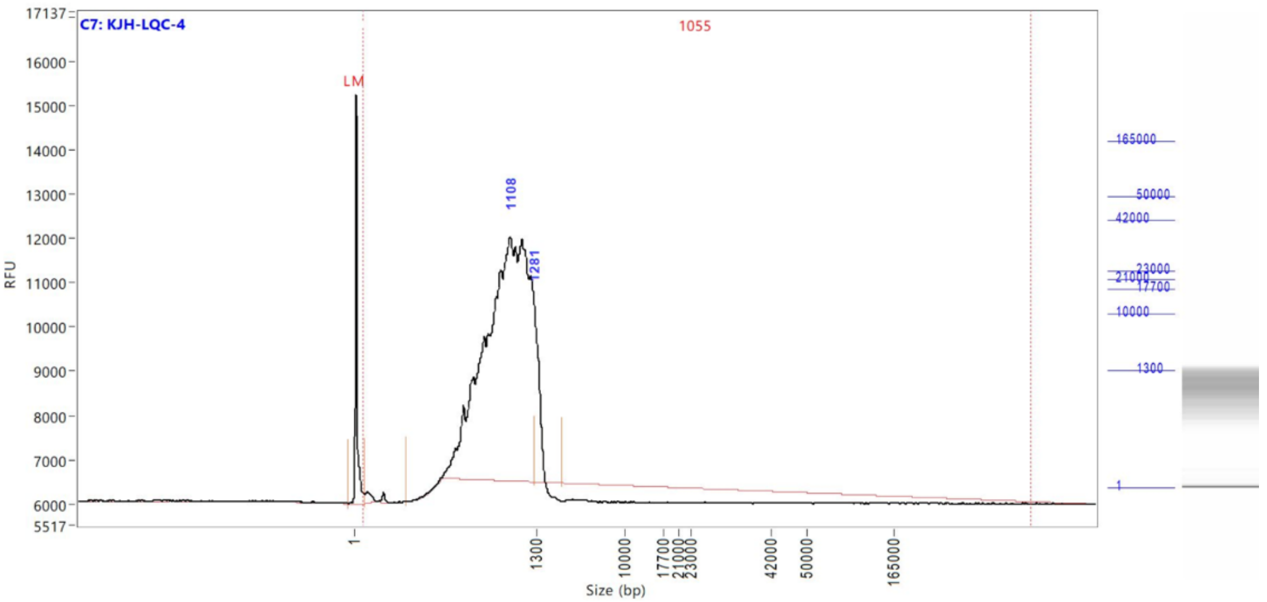

ID 5: 1 adult individual

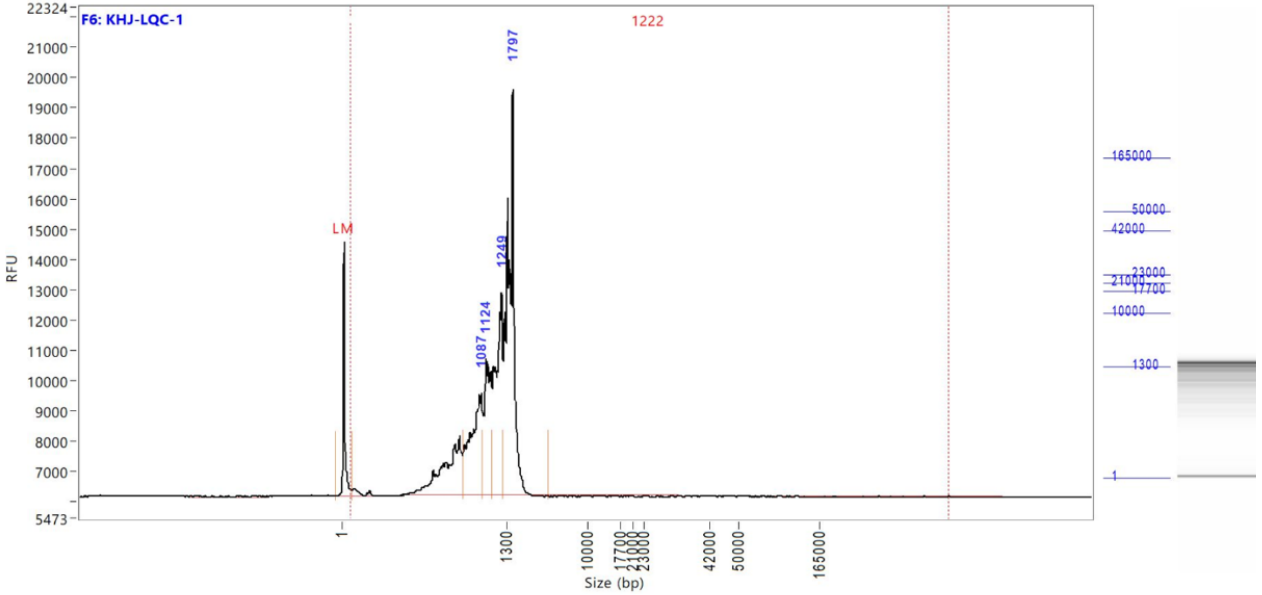

ID 6: 2 adult individuals

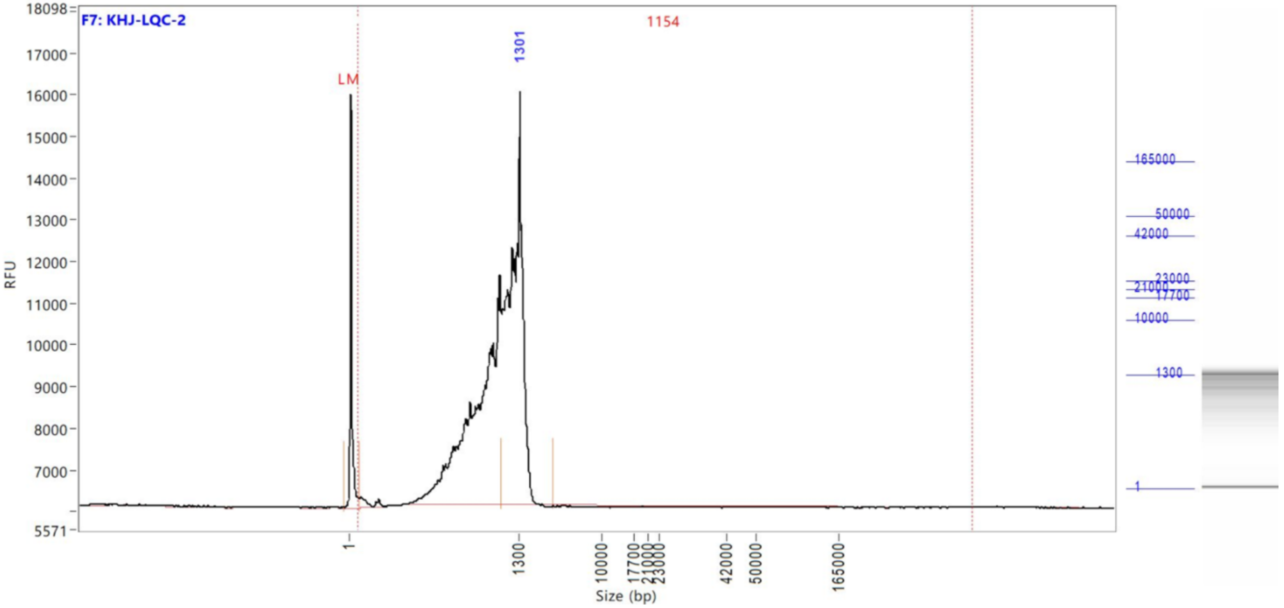

ID 7: 3 adult individuals

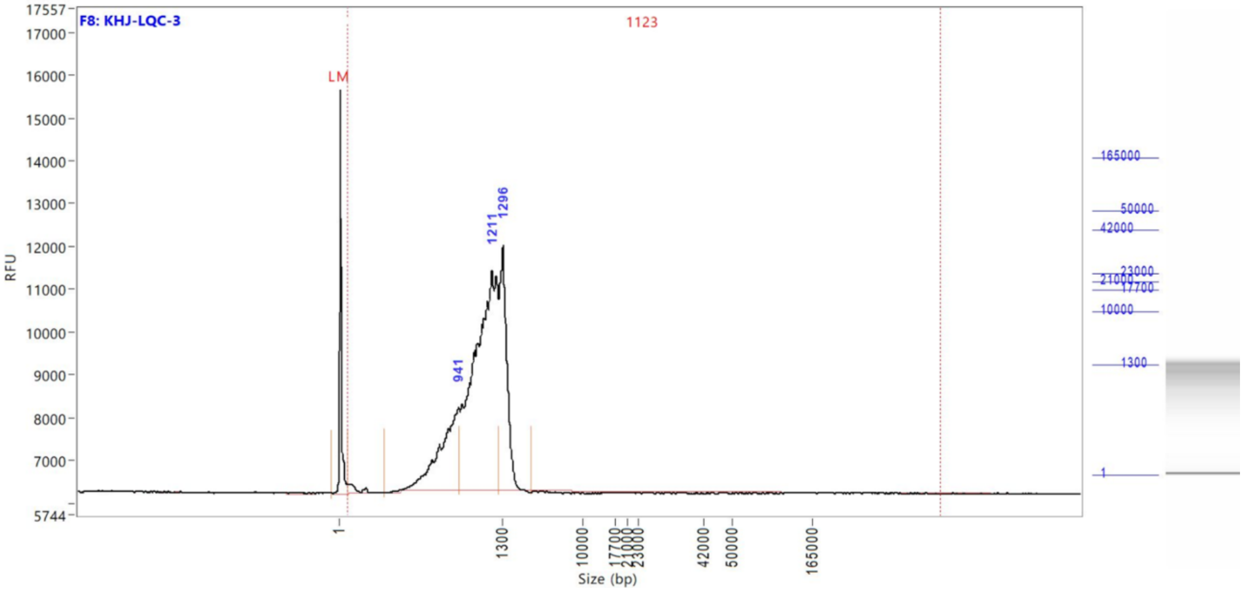

ID 8: 10 adult individuals

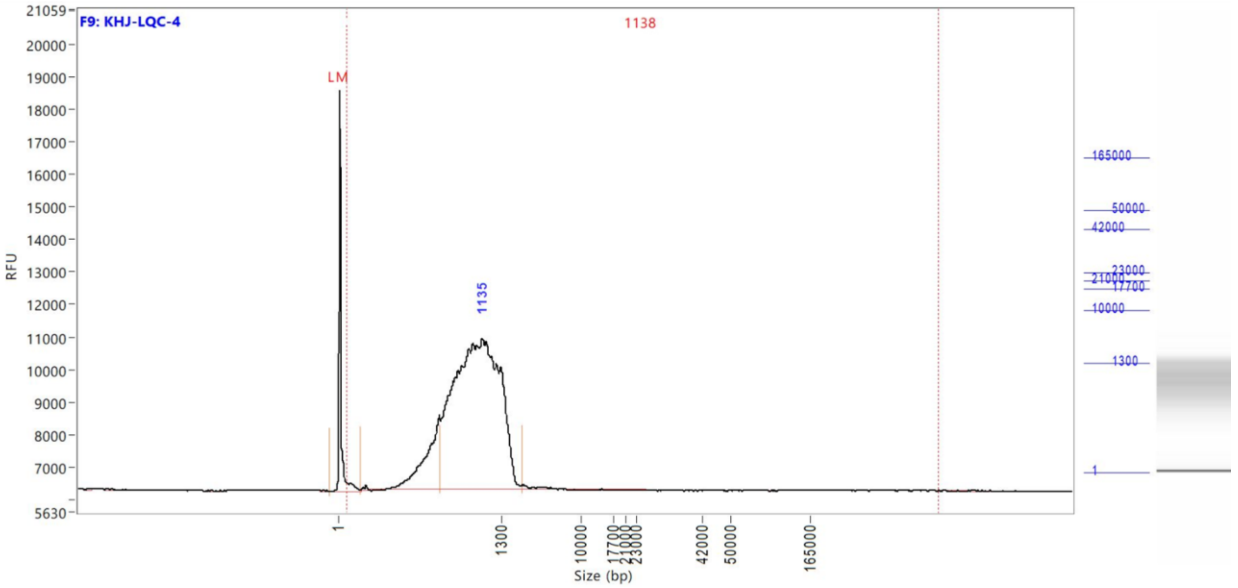

Figure S1 Electropherograms of cDNA/DNA Fragment-Size Distributions Obtained by Femto Pulse Electrophoresis
